# Training population selection and use of fixed effects to optimize genomic predictions in a historical USA winter wheat panel

**DOI:** 10.1007/s00122-019-03276-6

**Published:** 2019-01-24

**Authors:** J. Martin Sarinelli, J. Paul Murphy, Priyanka Tyagi, James B. Holland, Jerry W. Johnson, Mohamed Mergoum, Richard E. Mason, Ali Babar, Stephen Harrison, Russell Sutton, Carl A. Griffey, Gina Brown-Guedira

**Affiliations:** 1 Department of Crop and Soil Sciences, North Carolina State University, Raleigh, NC 27695 USA; 2 USDA-ARS Plant Science Research, North Carolina State University, Raleigh, NC 27695 USA; 3 Department of Crop and Soil Sciences, University of Georgia, Athens, GA 30602 USA; 4 Department of Crop Soil and Environmental Sciences, University of Arkansas, Fayetteville, AR 72701 USA; 5 Department of Agronomy, Louisiana State University, Baton Rouge, LA 70803 USA; 6 AgriLife Research, Texas A&M University, College Station, TX 77843 USA; 7 Department of Crop and Soil Environmental Sciences, Virginia Tech, Blacksburg, VA 24061 USA; 8 Agronomy Department, University of Florida, Gainesville, FL 32611 USA

## Abstract

**Key message:**

The optimization of training populations and the use of diagnostic markers as fixed effects increase the predictive ability of genomic prediction models in a cooperative wheat breeding panel.

**Abstract:**

Plant breeding programs often have access to a large amount of historical data that is highly unbalanced, particularly across years. This study examined approaches to utilize these data sets as training populations to integrate genomic selection into existing pipelines. We used cross-validation to evaluate predictive ability in an unbalanced data set of 467 winter wheat (*Triticum aestivum* L.) genotypes evaluated in the Gulf Atlantic Wheat Nursery from 2008 to 2016. We evaluated the impact of different training population sizes and training population selection methods (Random, Clustering, PEVmean and PEVmean1) on predictive ability. We also evaluated inclusion of markers associated with major genes as fixed effects in prediction models for heading date, plant height, and resistance to powdery mildew (caused by *Blumeria graminis* f. sp. *tritici)*. Increases in predictive ability as the size of the training population increased were more evident for Random and Clustering training population selection methods than for PEVmean and PEVmean1. The selection methods based on minimization of the prediction error variance (PEV) outperformed the Random and Clustering methods across all the population sizes. Major genes added as fixed effects always improved model predictive ability, with the greatest gains coming from combinations of multiple genes. Maximum predictabilities among all prediction methods were 0.64 for grain yield, 0.56 for test weight, 0.71 for heading date, 0.73 for plant height, and 0.60 for powdery mildew resistance. Our results demonstrate the utility of combining unbalanced phenotypic records with genome-wide SNP marker data for predicting the performance of untested genotypes.

**Electronic supplementary material:**

The online version of this article (10.1007/s00122-019-03276-6) contains supplementary material, which is available to authorized users.

## Introduction

Historical sets of phenotypic data generated by plant breeding programs allow for potential integration of existing phenotypic information into genomic selection approaches that increase genetic gain through reduced breeding cycle times (Hayes et al. 2009; Dawson et al. 2013; Crossa et al. 2013). The prediction of genomic estimated breeding values (GEBVs) for genotypes with limited or no available phenotypic data is central to genomic selection approaches. GEBVs are estimated using genome-wide marker profiles and prediction models based on training populations containing both phenotypic and genotypic information on a set of individuals (Meuwissen et al. 2001).

Many cultivars of wheat grown in the USA are developed by breeding programs located at land grant universities. There is interest in incorporating the historical data derived from these breeding programs into genomic selection. Public programs have a long tradition of germplasm exchange and collaborative testing of experimental lines prior to cultivar release. Breeders routinely enter advanced lines into collaborative testing programs grown in multiple environments throughout targeted wheat growing regions within the USA. The annual GAWN is an example of one such collaboration. Public university breeding programs located in eight southern states submit and evaluate advanced generation breeding lines in the GAWN, and phenotypic data are collected on multiple traits across environments within each state. The wealth of phenotypic data available for testing programs such as the GAWN could be combined with genotypic data to implement genomic selection. These data can also be leveraged to evaluate approaches to optimize predictive ability of genomic selection models.

One challenge of using historical datasets in genomic selection is their unbalanced nature because few entries are present in all years. This situation of unbalanced data makes it difficult to train the prediction model accurately. However, Bernal-Vasquez et al. (2017) showed that multiple year historical data sets can be effectively used to build genomic prediction models even with no overlap of entries across years. Incorporating high-density marker data in the analysis of unbalanced phenotypic data allows for estimation of a genomic relationship matrix that may be used for modeling genetic covariance for related entries tested in different years. Historical breeding trial data have been used for genomic prediction in an international wheat breeding program (Dawson et al. 2013) and in a rye (*Secale cereale* L.) breeding program (Auinger et al. 2016). Storlie and Charmet (2013) reported genomic selection predictive abilities between 0.2 and 0.5 for grain yield using an unbalanced set of elite winter wheat lines evaluated over 11 years at different locations in France. Rutkoski et al. (2012, 2015) showed the potential value of historical unbalanced data to predict GEBVs for resistance to Fusarium head blight (caused by *Fusarium graminaerum*) and stem rust (caused by *Puccinia graminis* f. sp*. tritici*) of wheat with predictive abilities between 0.1 and 0.4.

Determining the appropriate training population size and composition is critical for obtaining high prediction accuracies especially when we have genotypes from a large genetic group as candidates for training. Evaluation of effects of training population size on predictive ability has generally found that larger training populations are better (Arruda et al. 2015; Bentley et al. 2014; Cericola et al. 2017). However, the optimal training population size will vary with heritability of the trait, relatedness of the training population and prediction set, and population structure in the training and breeding populations. Studies have shown that properly accounting for population structure and genetic relatedness can increase prediction ability of genomic selection models (Crossa et al. 2014; Gou et al. 2014). In an empirical study of rice (*Oryza sativa* L.) and wheat, Isidro et al. (2015) demonstrated that the best method for training population selection depended on the genetic architecture of the trait and the level of population structure present. They determined that in the presence of strong population structure in rice, cluster analysis followed by stratified sampling strategies led to improved prediction accuracies. The effects on model predictive ability due to accounting for subpopulations in soft winter wheat germplasm require further evaluation. Other sampling strategies to optimize the training population have been proposed. Rincent et al. (2012) evaluated algorithms that minimize prediction error variance of the training candidate set and methods utilizing the generalized coefficient of determination (PEVmean and CDmean, respectively). These methods produced higher reliabilities than random selection of training populations with various sizes in two diversity panels of maize (*Zea mays* L.) inbreds. Akdemir et al. (2015) developed an alternative algorithm for efficient training population selection based on the minimization of PEVmean of individuals included in the test set and demonstrated that this method outperformed random training population selection in *Arabidopsis thaliana* L., wheat, rice, and maize. Therefore, we need to consider the optimization method suitable for our situation with wheat historical data for conducting genomic selection.

The choice of prediction methods is also one of the most important factors to determine the accuracy of genomic prediction. Of the statistical methods available to estimate GEBVs, one of the most utilized in genomic selection is ridge regression best linear unbiased prediction (RR-BLUP), a penalized regression method where all molecular marker effects are estimated from the training population and then used to predict GEBVs for individuals for which only genotypic data are available. RR-BLUP assumes that markers are random effects with common variance and all markers are equally shrunk toward zero by the same scaling factor (Meuwissen et al. 2001; Piepho 2009; Lorenz et al. 2011; de los Campos et al. 2013). Although widely used, the RR-BLUP approach estimates the effect of many loci with small effects simultaneously, resulting in an underestimation of the effects of major genes (Bernardo 2014). As an alternative to RR-BLUP alone, known major QTL can be modeled as fixed effects to account for variability due to major effect genes segregating in the germplasm studied (Spindel et al. 2016). Plant height and heading date of wheat are important traits influencing adaptation controlled by a combination of known major effect genes as well as genes of minor effect (Griffiths et al. 2009, 2012; Hanocq et al. 2007; Zanke et al. 2014). The dwarfing alleles *Rht*-*B1b* and *Rht*-*D1b* are major determinants of plant height and are each present at relatively high frequencies in winter wheat germplasm of the Eastern USA (Guedira et al. 2010). Allelic variation for important loci controlling heading date has also been reported in this winter wheat germplasm, including vernalization duration requirement related to the homeologous VERNALIZATION1 genes *Vrn*-*A1* and *Vrn*-*B1* and day length response related to the PHOTOPERIOD1 genes *Ppd*-*A1*, *Ppd*-*B1,* and *Ppd*-*D1* (Guedira et al. 2014, 2016). Each year the USDA-ARS Eastern Regional Small Grains Genotyping Laboratory evaluates entries in collaborative wheat breeding nurseries with marker assays that are predictive for alleles of these major plant height and heading genes, as well as assays for genes conferring resistance to disease and affecting end-use quality (https://triticeaetoolbox.org/wheat/). As a result, genetic information about the presence of major genes affecting agronomic traits is available to the breeder. However, the effect of these markers for causal loci on predictive ability of genomic selection models for plant height and heading date has not been reported.

Several other traits in wheat controlled by both major and minor effect loci segregate within breeding program germplasm for which trait-linked or casual markers remain unavailable. Identification of major effect loci for consideration as fixed effects could lead to improvement in predictive ability of genomic selection models. Markers linked to major QTL associated with important agronomic traits may be detected through association analyses. The effect of the markers identified by association analysis can then be considered as fixed effects when building genomic selection models while maintaining all other marker effects as random. Bernardo (2014) used simulation to examine the effect of modeling major QTL as fixed effects on prediction models and suggested that QTL explaining more than 10% of the variation associated with the trait should be included as model fixed effects. The entries in the GAWN provide an appropriate test case to evaluate the identification and inclusion of markers associated with resistance to wheat powdery mildew as fixed effects in genomic selection models for this trait. Although phenotypic variability for reaction to powdery mildew is present in the germplasm, the genetic basis of this variation is not known.

The primary objective of this study was to evaluate the use of a historical unbalanced data set to train genomic selection models in wheat. As selection of the appropriate training population is critical to obtaining accurate genomic predictions, we examined the effects of selecting training populations of different sizes using a strategy that considered population structure based on cluster analysis and strategies utilizing the PEVmean algorithm. In addition, we evaluated the effect of modeling known major genes for plant height and heading date and newly identified markers affecting resistance to powdery mildew as fixed effects in models for the respective traits. Overall, our goal was to determine how these factors affect the predictive ability of genomic selection models in this set of winter wheat germplasm.

## Materials and methods

### Plant material

A set of 483 soft red winter wheat elite lines in the F_8_ or later generations plus nine cultivars serving as checks were evaluated in field environments from 2008 to 2016. All lines were included in the phenotypic data analysis. The experiments were part of the annual GAWN cooperative testing program and represented elite germplasm from public breeding programs at the University of Arkansas (UA), University of Florida (UF), University of Georgia (UG), Louisiana State University (LSU), North Carolina State University (NCSU), Clemson University (CU), Texas A&M AgriLife Research (TAMU) and Virginia Polytechnic Institute and State University (VPI). The data set was balanced for individual years where the same set of genotypes was evaluated across different locations and unbalanced between years. The number of entries per year varied between 44 and 82, and the number of genotypes entered by each breeding program varied across the historical series (Table 1). Although entries developed at CU were evaluated in 2008 through 2012, the field evaluation was not conducted in CU. Also, the GAWN was evaluated at Farmersville, TX in all years; however, entries developed by the TAMU breeding program were submitted only in 2015 and 2016. Besides the check cultivars, the number of entries evaluated for grain yield and test weight in more than one year was 36. Overlap of entries evaluated for different traits across years is indicated in S. Table 1 and S. Table 2.

**Table 1:** Test year, entries per cooperating state breeding programs and total numbers of check cultivars and elite advanced line entries in the Gulf Atlantic Wheat Nursery from 2008 to 2016

Test year	Breeding programs								Check cultivars	Total by year
	UA	UF	UG	LSU	NCSU	CU	TAMU	VPI		
2008	12	7	12	12	12	12	0	12	3	82
2009	13	0	12	10	12	12	0	12	3	74
2010	12	1	12	11	12	12	0	12	3	75
2011	0	0	12	12	12	4	0	12	4	56
2012	12	1	12	11	12	12	0	12	4	76
2013	12	1	12	11	12	0	0	12	4	64
2014	5	0	7	6	6	0	0	10	4	38
2015	6	0	6	6	6	0	6	10	4	44
2016	4	0	6	7	9	0	6	10	3	45
Total by program	76	10	91	86	93	52	12	102		

### Phenotypic data collection and analyses

The GAWN was evaluated at one location in up to seven states per year from 2008 to 2016: Arkansas (Stuttgart or Marianna), Florida (Citra or Quincy), Georgia (Plains), Louisiana (Winnsboro), North Carolina (Kinston), Texas (Farmersville), and Virginia (Warsaw). Experimental designs at each environment were randomized complete block designs with two to three replications, although data for some traits were recorded on only one replication. Plot sizes across locations varied but were typical of yield trial plots for wheat in the region and were a minimum of 1.3 m wide and 3.1 m long. Data were obtained from four to seven locations annually, with an average of 5.9 locations per year over the nine seasons. Historical data consisted of plot-level data for grain yield and means for test weight, plant height, heading date and reaction to powdery mildew. Out of a total of 63 potential location-year combinations (environments, S. Table 3), data for grain yield were available for 49, test weight for 49, heading date for 53, plant height for 44 and powdery mildew resistance for 19.

During the 2015–2016 growing season, an additional experiment was conducted to collect data for heading date, plant height, and reaction to a natural epidemic of powdery mildew in a common environment (this experiment is not included in Table 1). A total of 391 lines for which adequate seed was available were grown in Raleigh, North Carolina, in a randomized complete block design experiment with two blocks. Four common checks were repeated every 80 rows. Plots were 1-m rows spaced 30 cm apart. Heading date was recorded as day of year when 50% of the plants in a plot were with the spike emerged (Zadoks growth stage 55, Zadoks et al. 1974). Plant height was recorded as the distance in cm from soil level to the tip of the spike, excluding awns. Powdery mildew reaction was recorded using a 0–9 scale, incorporating both height and intensity of conidia in the canopy. A value of 0 indicated complete absence of conidia in the canopy, and a value of 9 indicated conidia throughout the canopy and on the flag leaf. These data were included in the analysis to determine if addition of measurements from a common environment would improve model predictive ability.

The following linear mixed model was utilized for the analysis of grain yield, for which plot-level data were available:

$${\text{y}}_{ijkl} = \mu + {\text{Y}}_{i} + {\text{L}}_{j} + {\text{B}}({\text{YL}})_{ijk } + {\text{YL}}_{ij } + {\text{G}}_{l} + {\text{YG}}_{il } + {\text{LG}}_{jl} + {\text{YGL}}_{\text{ijl}} + \varepsilon_{ijkl}$$

where y_*ijkl*_ was the phenotypic observation of genotype *l* in the *i*th year in the *j*th location in the *k*th block, was the overall mean, Y_*i*_ was the year effect, L_*j*_ was the location effect, B(YL)_*ijk*_ was the block effect nested within year and location, G_*l*_ was the genotypic effect, YL_*ij*_, YG_*il*_, LG_*jl*_, YLG_*ijl*_ were the interaction terms representing year by location, genotype by year, genotype by location and genotype by year by location, respectively, and _*ijkl*_, represented the residual term. The overall mean and the genotypic effects were considered fixed and all the remaining terms random. The error variance component for this trait was allowed to be heterogeneous across environments (Y_*i*_ and L_*j*_ combinations), but IID within environments. All other random effects had independent, identical normal distributions with mean zero and common variance for all effects of a given factor.

Only a single mean value for each line-environment combination was available for test weight, heading date, plant height and powdery mildew resistance. Therefore, the following linear mixed model was fit to the data for test weight, heading date, and plant height:

$${\text{y}}_{ijl} = \mu + {\text{Y}}_{i} + {\text{L}}_{j} + {\text{YL}}_{ij } + {\text{G}}_{l} + {\text{YG}}_{il } + {\text{LG}}_{jl} + \varepsilon_{ijl} ,$$

where terms are identical to the grain yield model, but block and YGL interaction effects are not included in the model, and the residual effect represents a confounded term of YGL interaction and residual effects of within-environment mean values. Furthermore, for these traits, a homogeneous residual error variance term was fitted: *ε* ~ IID *N*(0, $\sigma_{e}^{2}$) due to the lack of replications within each environment.

Finally, powdery mildew was measured in only 19 environments, insufficient to estimate the all the variance parameters in the previous model. Therefore, for powdery mildew only, the effects of year and location were combined into a single term called environment:

$${\text{y}}_{ijl} = \mu + E_{ij} + {\text{G}}_{l} + \varepsilon_{ijl}$$

The models were implemented using ASReml-R (Butler et al. 2009). Best linear unbiased estimates (BLUE) of each genotype were calculated as the estimated genotypic effect plus overall mean and used as the values of a dependent variable in a genomic prediction model. Estimates of broad sense heritability on a plot basis for each trait were calculated using a statistical model similar to the model previously described but with the overall mean as a fixed effect and all other terms random. The residual variance was uniform across environments in the analysis of each trait. The broad sense heritability on a per plot basis of each trait was computed according to Holland et al. (2003) as:

$$H^{2} = \frac{{\sigma_{g}^{2} }}{{\sigma_{g}^{2} + \sigma_{gy}^{2} + \sigma_{gl}^{2} + \sigma_{gyl}^{2} + \sigma_{e}^{2} }}$$

where $\sigma_{g}^{2}$ represents genotypic variance, $\sigma_{gy}^{2}$, $\sigma_{gl}^{2}$, $\sigma_{gyl}^{2}$, are the variance components due to the genotype by year, genotype by location and genotypes by location by year, respectively, and $\sigma_{e}^{2}$ is the variance associated with the residual term.

### Genotypic data

Genotyping by sequencing (GBS, Elshire et al. 2011) using the protocol described by Poland et al. (2012) was conducted for 467 of the 492 lines that were phenotyped, including elite genotypes and check cultivars. Seeds of 25 of the older lines were no longer available. Ninety-six individual samples were pooled into a single library, and each library was sequenced on an Illumina HiSeq 2500. Single nucleotide polymorphism (SNP) calling on raw sequence data was done with Tassel5GBSv2 pipeline version 5.2.35 (Glaubitz et al. 2014) with alignment to the International Wheat Genome Sequencing Consortium (IWGSC) RefSeq1.0 assembly (https://wheat-urgi.versailles.inra.fr/Seq-Repository/Assemblies) using Burrows-Wheeler aligner (BWA) version 0.7.12 (Li and Durbin 2009). SNP with ≤ 50% missing data, ≥ 5% minor allele frequency and ≤ 10% of heterozygous calls per marker locus were retained and imputation performed using Beagle in the R package Synbreed (Wimmer et al. 2012; Browning and Browning 2016). After imputation, redundant SNP less than 64 bp apart were removed from the data set. The final number of SNP utilized for analyses was 34,095.

All lines were evaluated with kompetitive allele specific PCR (KASP) assays diagnostic for genes having major effects on plant height and heading date that are routinely used for characterization of lines in southeastern breeding programs. These included assays associated with plant height (*Rht*-*B1* and *Rht*-*D1*), vernalization requirement duration (*Vrn*-*A1* and *Vrn*-*B1*), and photoperiod (*Ppd*-*A1*, *Ppd*-*B1*, *Ppd*-*D1*). In addition, assays that detect alien translocations including the *t1AL:1RS* and *t1BL:1RS* translocations from rye, the *t2BS:2GS·2GL:2BL* translocation from *Triticum timopheevii* and the translocation *t2AS:2NS* from *Triticum ventricosum* were screened (S. Table 4). These data are available for download at the T3 database (https://triticeaetoolbox.org/wheat/).

Genomic information was organized in a matrix where data for genotypes were organized in rows and marker scores for the 34,095 SNP from GBS plus the KASPs assays were arranged in columns. All marker data were coded 0, 1 or 2 according to the number of copies of the minor allele. Principal components analysis of this matrix was implemented with the prcomp function in R version 3.3.1 (R Core Team 2016).

### Training population and validation set

The predictive ability of genomic selection models for each of the five traits was measured as mean Pearson correlations between BLUEs and GEBVs across 50 iterations of cross-validation. For cross-validation, we selected 50 different validation sets of size 60 (the average number of unique entries evaluated each year) as a random sample of genotypes from the 467 wheat lines with phenotypic and genotypic data. The same 50 validation sets were consistently utilized throughout the experiment to ensure accurate comparisons between different genomic selection methods. Data for each validation set were masked when we built a prediction model.

The effect of training population size on the predictive ability of the genomic selection model was assessed using seven different population sizes (50, 100, 150, 200, 250, 300, and 350). For each population size, we implemented four approaches for comparison of the training population selection:

#### Random

Random training population selection was utilized as the reference method to compare with the other three methods of training population optimization. For this method, a random sample of individuals was selected as training population for each validation set according to the size of the training population.

#### Clustering

Benson et al. (2012) demonstrated that chromosomal translocations involving related genomes segregating in soft winter wheat germplasm were associated with subpopulation differentiation. In this study, we identified population structure associated with the translocation *t2BS:2GS·2GL:2BL*. Thus, we attempted to optimize the training population design by assigning the same proportion of individuals with and without the translocation in the training population and in each validation set. For each validation set randomly selected from the 467 genotypes, the proportion of individuals with the translocation was determined. The remaining individuals (potential training population candidates) were split based on the presence or absence of the translocation, and sample sizes for each group were chosen so that the translocation group frequencies were the same between training and validation sets. A random sample of the individuals required in each translocation set was selected and merged to form the training population. The same procedure was utilized across the 50 different validation sets and for each training population size.

#### PEVmean

This approach utilized a training population optimization algorithm for each validation set that minimized the mean prediction error variance (Rincent et al. 2012; Akdemir et al. 2015). The PEVmean algorithm used genomic information from all genotypes to measure the reliability of the GEBVs for individuals in the validation set. An optimal training population from all genotypes available was selected to minimize the mean prediction error variance in the validation set. We used the approach suggested by Akdemir et al. (2015) for an efficient approximation to the prediction error variance using the first 100 principal components of the genotypes to estimate the genomic relationship matrix. The PEVmean strategy was implemented using the function GenAlgForSubsetSelection from the R package STPGA with optimality criterion: “PEVMEAN” (Akdemir 2016). Principal components were estimated from genotypic data, and the first 100 principal components were chosen for error variance estimation. The best training population for each of the 50 validation sets for each of the seven different population sizes was selected after 300 iterations of the genetic algorithm parameter, while other parameters in the function were set with default values.

#### PEVmean1

This was a modification of the previously described PEVmean training population optimization design whereby we identified the optimum training population that minimized the prediction error variance for every individual in the validation set. This procedure was implemented in the R package STPGA as described above for each individual genotype in the validation set with the goal of identifying the best training population to predict a single genotype. In PEVmean1, the same GenAlgForSubsetSelection function was used in the STPGA package with only one genotype in the validation set. Thus, we obtained 60 optimized training sets for each cross-validation step that were used to independently predict each genotype in the validation set. The procedure was repeated for each of the 50 validation sets and for the different training population sizes.

### Genomic selection and association analysis

The RR-BLUP model described by (Meuwissen et al. 2001; Piepho 2009) was used to estimate GEBV. The model was specified as follows:

$$y = \varvec{X}\beta + \varvec{Z}u + e$$

where *y* was a vector of BLUEs for each wheat genotype obtained for one trait, *β* was a vector of fixed effects which included the overall mean and fixed covariates (major gene and association mapping markers), ***X*** and ***Z*** were the design matrices for fixed and random effects, *u* was a vector of random marker effects, and *e* was a vector representing the residual terms. The variance–covariance structure associated with the random term was *u* ~ *N* (0, *I*$\sigma_{u}^{2}$) and for the residual term was *e* ∼ *N* (0, *I*$\sigma_{e}^{2}$). The estimates of *u* were obtained from the mixed.solve function using the package rrBLUP in R (Endelman 2011).

The impact of incorporating markers previously associated with one or more major effect locus as fixed effects in the genomic selection model was measured as the change in predictive ability of models with and without markers as fixed effects in the model. When a marker entered the model as a fixed effect, the same marker was removed from the matrix of markers as random effects. Markers predictive of the dwarfing alleles *Rht*-*D1b* and *Rht*-*B1b* were used as fixed effects in models to predict plant height. Polymorphisms in exon 4 of *Vrn*-*A1* (Díaz et al. 2012; Yan et al. 2015; Guedira et al. 2016) and the first intron of *Vrn*-*B1* (Guedira et al. 2014, 2016) associated with differences in duration of vernalization requirement and heading date in winter wheat were included as fixed effects in models to predict heading date. In addition, an assay predictive of the insensitive allele *Ppd*-*D1a* of the major photoperiod locus on chromosome 2D (Beales et al. 2007) was also included as a fixed effect in the models to predict heading date.

Association analysis using a mixed linear model implemented in the R package GAPIT (Lipka et al. 2012; Tang et al. 2016) was performed to identify significant markers for grain yield, test weight and powdery mildew resistance. To avoid bias in the calculation of predictive ability for the genomic selection model, the identification of markers to use as fixed effects was based on genome-wide association study (GWAS) specific to each training set of lines only. The first three principal components and the genomic relationship matrix were utilized for GWAS to account for population structure and degree of relatedness between individuals in the population to avoid spurious associations (Yu et al. 2006). Markers were declared significant based on the Bonferroni corrected *p* value at *α* = 0.01. We planned to use the detected markers as fixed effects in prediction models.

### Comparing effects on genomic selection prediction accuracy

Pairwise comparisons of predictive ability for different training population optimization methods and for the addition of markers as fixed effects in genomic selection models were performed for different training population sizes and traits considered in the analysis. The effect of different prediction methods was tested using a one-way ANOVA using the model:

$$r = \mu + Treatment + error$$

where *r* is the vector of predictive abilities with a *z*-transformation to avoid lack of normality in the error term, *µ* is the overall mean predictive ability, *Treatment* is the fixed effect of different training population optimization methods or the different models including fixed effects and *error* represents the residual term in the model.

## Results

### Phenotypic summary

Nine years of phenotypic data for grain yield, test weight, heading date, plant height, and powdery mildew resistance from a historical data set of the GAWN were curated and analyzed. The total number of data points (GxLxY) available for analysis were 7028 for grain yield, 5075 for test weight, 4861 for heading date, 4780 for plant height and 2246 for powdery mildew reaction. The effect of genotype was significant for all traits analyzed (Table 2). Genotype by environment interactions was significant for all traits except plant height, for which genotype by year and genotype by location interactions were not significant. Broad sense heritability on a per plot basis was low for grain yield (0.14), intermediate for test weight (0.28), and higher for heading date, plant height and powdery mildew resistance (0.49–0.57). Line means for grain yield ranged from 0.42 to 8.27 Mg ha^−1^, with a mean of 4.24 Mg ha^−1^.

**Table 2:** Summary of phenotypic information for grain yield, test weight, heading date, plant height and powdery mildew resistance, including number of environments where each trait was evaluated, number of data points for the analysis of each trait, descriptive statistics, variance components estimates, and broad sense heritability estimate calculated on a per plot basis

	Traits				
	Grain yield	Test weight	Heading date	Plant height	Powdery mildew
	Mg ha^−1^	kg m^−3^	Days	cm	0–9 scale
No. environments	49	49	53	44	19
No. data points	7028	5075	4861	4780	2446
Minimum	0.42	41.70	63.00	53.34	0.00
Mean	4.24	57.05	105.56	88.34	2.16
Maximum	8.27	65.80	131.00	137.16	9.00
Standard deviation	1.30	6.60	11.91	11.07	2.01
Variance components estimates					
Location (L)	52.60*	3.12*	37.95*	3.24*	0.60*
Year (Y)	53.82	0.97	43.26*	2.82*	
YL	142.09*	5.41*	21.67*	4.05*	
Rep (YL)	3.76*	0.07*	0.08*	0.15*	0.10*
Genotype (G)	19.84*	1.07*	14.42*	4.85*	1.66*
GY	6.54*	0.41*	1.26*	0.13	0.62*
GL	26.24*	0.32*	5.39*	0.06	
GYL	50.71*	1.26*	4.12*	1.10*	
Residual	39.65	0.80	1.45	2.42	1.03
Heritability	0.14	0.28	0.54	0.57	0.49

*Significant at *α* = 0.05

### Genotypic data and population structure

The final marker data set consisted of 34,095 bi-allelic SNP markers from GBS and 12 KASP markers on the panel of 467 genotypes. The level of polymorphism detected by GBS varied for each genome with 13,190 SNP detected in the A genome, 16,522 SNP detected in the B genome, and 4575 SNP detected in the D genome (S. Table 5). Evaluation with KASP markers determined that most lines in the population were semi-dwarf having either *Rht*-*D1b* (74%) or *Rht*-*B1b* (20%) while the remaining 6% did not possess either semi-dwarfing allele. Twenty-one percent of lines possessed a winter allele at *Vrn*-*A1* associated with short vernalization duration requirement, 28% possessed the short vernalization requirement allele at *Vrn*-*B1* and 63% of the lines had the *Ppd*-*D1* allele for photoperiod insensitivity (Table 5).

**Fig. 1:**
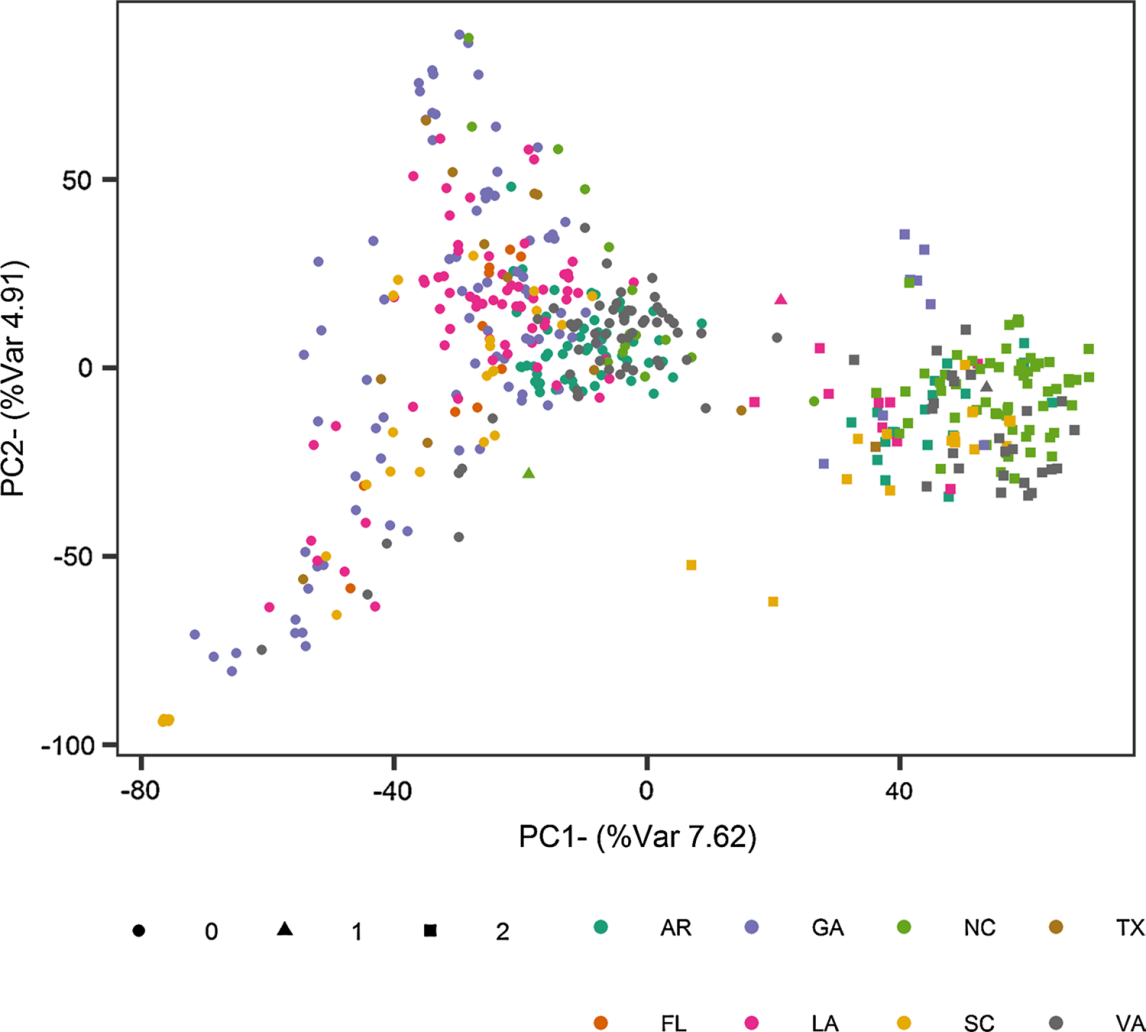
Scatter plot of the first two principal components from analysis of 467 winter wheat genotypes based on the full data set of 34,107 markers. Points are color coded according to the origin of genotypes: AR, University of Arkansas; FL, University of Florida; GA, University of Georgia; LA, Louisiana State University; NC, North Carolina State University; SC, Clemson University; VA, Virginia Tech; TX, Texas A&M AgriLife Research. Different shapes represent the number of copies of the allele of SNP marker IWA8068 located in the *t2BS:2GS·2GL:2BL* translocation from *T. timopheevii*. Percentages in each axis represent the proportion of variance explained by each principal component

Two distinct clusters were observed on the first principal component axis that were related to the presence of the *t2BS:2GS·2GL:2BL* translocation derived from *T. timopheevii* (Fig. 1). The correlation between the first principal component and the scores of the diagnostic KASP assay based on SNP marker IWA8068 that resides in the translocation was 0.89. Of the 467 genotypes in the data set, 145 possessed the translocation. Analysis of the marker matrix identified many chromosome 2B SNP having alleles exclusive to genotypes possessing the translocation. Although a separation of lines into two clusters based on the presence/absence of the alien translocation was observed, the first principal component explained less than 8% of the total variation in the data set based on markers. Within the cluster of genotypes lacking the translocation, there was some separation of lines developed by programs in the more southern states of GA and SC, compared to lines developed in the Mid-Atlantic programs in VA and NC (Fig. 1). Lines from GA were noticeable for their diversity on both the first and the second principal components.

### Effect of training population size and optimization on predictive ability of genomic selection models

**Fig. 2:**
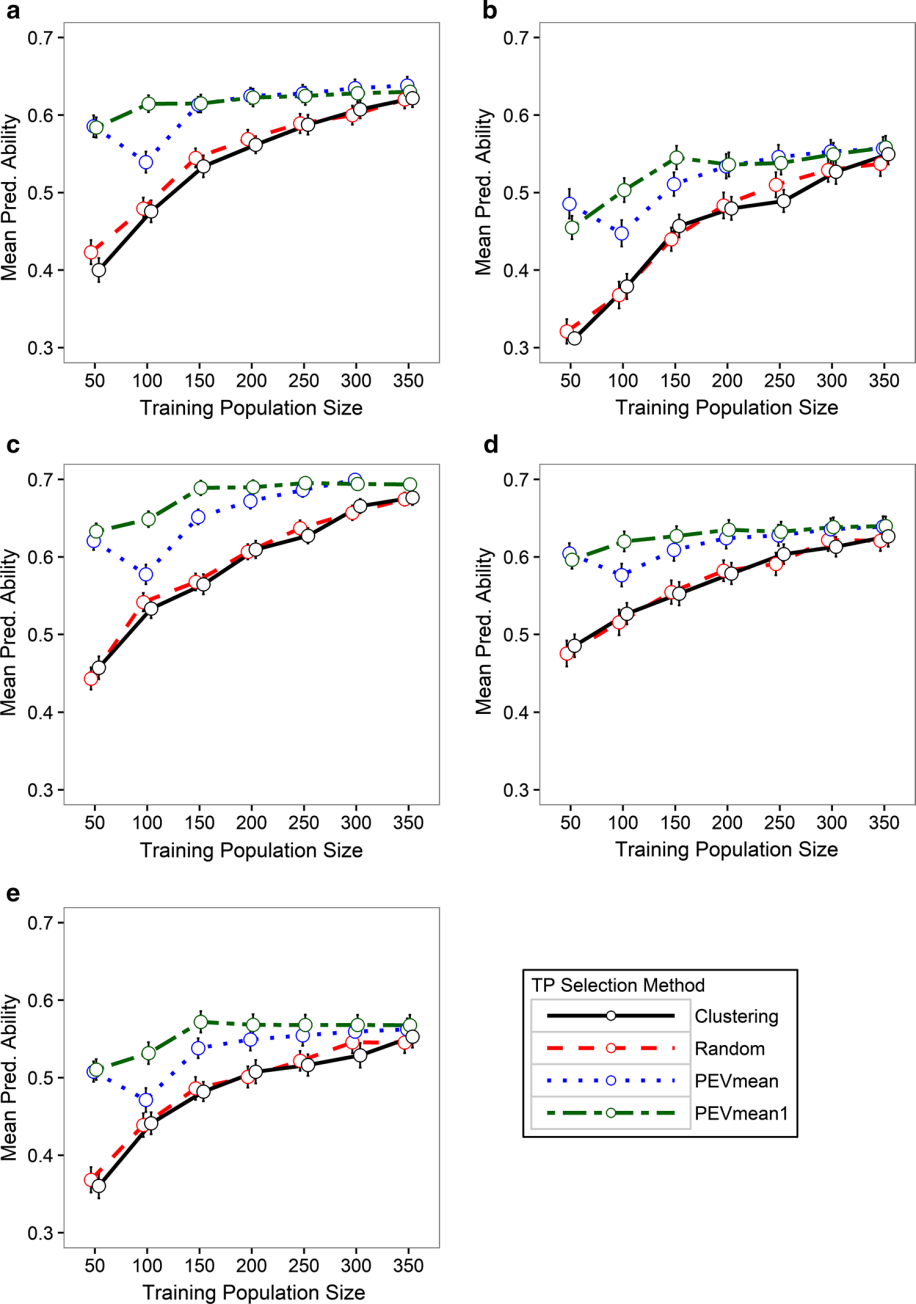
Comparison of mean predictive ability (Mean Pred. Ability) for grain yield (**a**), test weight (**b**), heading date (**c**), plant height (**d**) and powdery mildew resistance (**e**) using four training population optimization methods: Clustering (Weighted proportion of translocation *t2BS:2GS·2GL:2BL* in the training population and validation set), PEVmean (training population selected by minimization of the PEV mean in the validation set), PEVmean1 (training population selected by minimization of the PEV of each individual in the validation set) and Random (random training population selection). All methods were evaluated for seven different training population sizes. Error bars represent ± one standard error of the mean

Predictive ability of genomic selection models varied with training population sizes and optimization methods (Fig. 2). Mean predictive abilities from cross-validation for grain yield (0.40–0.64) and test weight (0.31–0.56) were particularly encouraging given the importance of these traits as primary selection goals in wheat breeding. Slightly higher predictive abilities were observed for heading date (0.44–0.70) and plant height (0.47–0.64). Predictive ability for powdery mildew resistance ranged from 0.36 to 0.57.

The effect of incorporating Raleigh 2016 as a common environment for measurement of plant height, heading date and powdery mildew resistance increased the mean predictive ability of heading date when selecting the training population at random and using the PEVmean selection criteria (Table 3). For plant height and powdery mildew resistance, the effect of data from a common environment on the overall mean predictive ability of the models was marginal or null. Based on observed improvements for heading date predictions, the Raleigh 2016 data were retained in subsequent analyses.

**Table 3:** Mean predictive ability after 50 cycles of cross-validation using two TP optimization methods (Random and PEVmean) for heading date, plant height and powdery mildew resistance calculated and averaged across seven TP sizes. Genomic selection models used only phenotypic data available from the historical series or incorporated phenotypic data from a common environment (Raleigh 2016) along with the historical series

	All locations	Historical data
Heading date		
Random	0.59**	0.53
PEVmean	0.66**	0.58
Plant height		
Random	0.57	0.57
PEVmean	0.62	0.62
Powdery mildew resistance		
Random	0.48	0.48
PEVmean	0.53	0.52

**Significant at *α* = 0.01

Model predictive ability generally increased as the size of the training population increased. In the Random and Clustering methods, the magnitude of increase in predictive ability for all the traits was greatest between training population size 50–250, while increases in the population sizes over 250 individuals did not have a significant impact. When using methods based on minimizing the PEV, an increase in the training population size beyond 150 did not increase predictive ability for the all traits except heading date. Increasing training population size beyond 200 did not increase predictive ability for heading date. It was noteworthy that for grain yield, when using training population selection PEVmean and PEVmean1, there was a high predictive ability even when the training population size was 50, and there was no substantial increase in predictive ability for any other training population size. Predictive ability for training population optimization method PEVmean at training population size of 100 was lower than training population size 50 for all traits but still outperformed training population selection methods Random and Clustering.

Optimization of the training population by methods that minimize the prediction error variance of the individuals in the validation set (PEVmean, PEVmean1) outperformed models that used a purely random approach (Random) or a combination of clustering and random sampling (Clustering, Fig. 2). This trend was observed for all traits and at all training population sample sizes. Significant differences between optimization methods were found for training population sizes between 50 and 300, while for training population size 350, the different methods tended to converge. PEVmean and PEVmean1 had predictive abilities that were significantly higher (*p* < 0.05) than Clustering and Random for sample sizes from 50 to 200 genotypes. Although, PEVmean and PEVmean1 performed better at training population sample sizes from 250 to 350, in most cases the difference in predictive ability with the other methods were not significant.

### Effect of fixed effect markers on predictive ability

Investigation into the impact on genomic selection model predictive ability through inclusion of markers for major genes as fixed effects versus random effects utilized two training population selection methods, PEVmean and Random, for the seven different training population sizes. For heading date and plant height, markers in previously identified major genes were used as fixed effects. No significant markers were identified for grain yield and test weight after association analyses, so models having fixed effects derived from major QTL were not evaluated for these traits. The most significant marker trait association for powdery mildew resistance varied depending on the independent training set utilized for association analysis. However, for all training sets, the most significant markers were in the terminal region of the long arm of chromosome 7A indicative of a gene of major effect on powdery mildew resistance in this region (S. Table 6). For these analyses, only the single most significant marker identified in each independent association analysis was utilized as a fixed effect for prediction of the respective validation set to make the number of fixed effects in the model uniform in all cross-validation sets.

Overall, we observed that including markers associated with major effect genes or QTL as fixed effects increased the predictive ability similarly for both TP selection methods when compared with models without fixed effects (S. Fig. 1, S. Fig. 2, S. Fig. 3). Results based on the random model with and without fixed effects are shown in Table 4 for different traits, markers, and TP sizes. Markers for heading date utilized as fixed effects were based on polymorphisms in the *Vrn*-*A1*, *Vrn*-*B1,* and *Ppd*-*D1* loci that are known to be important determinants of flowering time in wheat. The addition of the marker for *Vrn*-*A1* had the largest impact on predictive ability when markers as fixed effects were evaluated individually (Table 4). Using a combination of markers in the *Vrn*-*A1*, *Vrn*-*B1,* and *Ppd*-*D1* genes, the predictive ability increased with all population sizes and was significantly different from models that considered one marker only. However, the impact of adding markers as fixed effects in the model decreased as training population size increased. When training populations contained 300 or more individuals, the only models that differed from the random model (no marker as fixed effect) were those that included multiple markers. Given a training population size of 50, an average of 10% increase in model predictive ability was observed when markers in the *Vrn*-*A1*, *Vrn*-*B1,* and *Ppd*-*D1* genes were included as fixed effects in the model, compared with the random model. The improvement in predictive ability when adding markers as fixed effect was 3% when the training population size was 350. Genomic selection models for plant height included genotypes at SNP in the reduced height genes *Rht*-*B1* and *Rht*-*D1* indicative of the semi-dwarfing alleles. The effect of the *Rht*-*D1b* allele in the genomic selection models was greater than that observed for *Rht*-*B1b* (Table 5). The genomic selection models that included alleles at *Rht*-*B1* as a fixed effect did not increase predictive ability across different training population sizes, whereas the model with alleles at *Rht*-*D1* treated as fixed effects significantly increased predictive ability over all training population sizes (Table 4). Including both diagnostic markers as fixed effects in the model produced increases in the predictive ability compared with models having only *Rht*-*D1*. Predictive ability of models having *Rht*-*D1* and *Rht*-*B1* ranged from 9 to 17% higher compared with the models without markers as fixed effects across the different training population sizes evaluated.

**Table 4:** Comparison of mean predictive ability across 50 cycles of cross-validation for heading date, plant height and powdery mildew resistance according to genomic selection models having no marker as fixed effects with models that consider the addition of trait associated markers as fixed effects. Analyses used the Random training population selection method and different training population sizes. Combinations of diagnostic markers for loci associated with heading date (*Ppd*-*D1*, *Vrn*-*A1*, *Vrn*-*B1*) and plant height (*Rht*-*B1*, *Rht*-*D1*) were utilized, while for powdery mildew resistance the most significant SNP detected in GWAS for each validation cycle was utilized as the fixed effect

	Training population size						
	TP050	TP100	TP150	TP200	TP250	TP300	TP350
Heading date							
No fixed marker	0.46	0.53	0.56	0.61	0.63	0.67	0.68
*Ppd*-*D1*	0.42*	0.54	0.58	0.62	0.64	0.68	0.69
*Vrn*-*A1*	0.51**	0.58**	0.60**	0.63*	0.65*	0.68	0.69
*Vrn*-*B1*	0.49	0.56	0.59	0.63	0.64	0.67	0.68
*Vrn*-*A1_Ppd*-*D1*	0.51**	0.59**	0.62**	0.65**	0.67**	0.70**	0.70**
*Vrn*-*A1_Vrn*-*B1_Ppd*-*D1*	0.56**	0.61**	0.64**	0.67**	0.68**	0.71**	0.71**
Plant height							
No fixed marker	0.49	0.53	0.55	0.58	0.60	0.61	0.63
*Rht*-*B1*	0.47	0.52	0.55	0.58	0.60	0.61	0.62
*Rht*-*D1*	0.56**	0.60**	0.62**	0.64**	0.66**	0.67**	0.68**
*Rht*-*B1_Rht*-*D1*	0.59**	0.64**	0.67**	0.69**	0.71**	0.72**	0.73**
Powdery mildew resistance							
No fixed marker	0.36	0.44	0.48	0.51	0.52	0.53	0.55
Most significant SNP	0.42**	0.50**	0.53**	0.56**	0.56**	0.57**	0.60**

*, **Significantly different from the no fixed marker model at *α* = 0.05 and *α* = 0.01, respectively

**Table 5:** Allele frequency, SNP position, and mean allelic effect for trait associated markers utilized as fixed effects for heading date and plant height

Trait	Locus	Chromosome	Position^a^ (Mbp)	Freq. of early/dwarf allele	Dwarf/early allele	Reference allele	Mean effect^b^	Std deviation^b^
Plant height (cm)	*Rht*-*B1*	4B	30.86	0.20	T	C	− 3.78	0.14
	*Rht*-*D1*	4D	18.78	0.74	T	G	− 5.38	0.15
Heading date (days)	*Vrn*-*A1*	5A	587.42	0.21	C	T	− 1.37	0.14
	*Vrn*-*B1*	5B	573.81	0.18	C	G	− 1.28	0.20
	*Ppd*-*D1*	2D	33.96	0.63	Deletion	Non-deleted	− 0.91	0.14

^a^Position of SNP in the International Wheat Genome Sequencing Consortium (IWGSC) RefSeq1.0 assembly

^b^Calculated as the average effect of the marker estimated from 50 different training populations of size 350 selected at Random to predict GEBVs in each of the 50 validation set for a model that included all diagnostic markers associated with the trait simultaneously

For powdery mildew resistance, we used the most significant SNP marker, detected by association analysis in each training set. Each time the GWAS was conducted, the data for individuals in the 50 different validations sets was masked to avoid biases in the estimation of the marker effect on predictive ability. The six different SNP markers selected as fixed effects are detailed in S. Table 6. The number of times that an individual SNP was declared the most significant in the 50 independent GWAS evaluated ranged from 1 to 25. The effect on model predictive ability of using the single most significant SNP detected in each independent GWAS as a fixed effect was significant for all training population sizes compared with models without fixed effects (Table 4). Given a training population size of 50, an average of 14% increase in predictive ability (from 0.36 to 0.42) was observed when the most significant SNP was included as a fixed effect in the model, compared with the random model. The improvement in predictive ability when adding the SNP as fixed effect was 8% when the training population size was 350 (0.55–0.60).

## Discussion

Questions concerning material to be utilized in the training population and how to optimize prediction ability of the model based on the germplasm available arise when breeders integrate genomic selection into an ongoing cultivar development program. This study reported the utility of a historical unbalanced data set for elite wheat lines from eight public breeding programs evaluated across the southeastern USA for genomic selection. These public programs collaborate to develop commercial cultivars of soft red winter wheat for the region, thus the study provides valuable empirical results on the use of genomic selection to predict the average genotypic value of genotypes across a wide area.

Overall, cross-validation results from this study were encouraging regarding the use of unbalanced historical data for genomic selection predictions, even for highly polygenic and complex traits like grain yield and test weight. The mean predictive ability for grain yield was 0.64 for a training population size of 350 individuals while using the training population optimization method PEVmean. The model predictability from our cross-validation results for grain yield in wheat were higher than the 0.40–0.50 reported by of Storlie and Charmet (2013), who used historical unbalanced data of 318 lines grown over an 11-year period in France and the 0.28–0.45 reported by Poland et al. (2012) in a panel of 254 wheat lines evaluated in Mexico during 2010.

While grain yield is a primary goal for improvement in wheat cultivar development programs, it is not the only target as quality parameters and resistance to abiotic and biotic stress are also of paramount importance. For the onetime cost of genotyping, the marker information is available to predict any trait, including complex traits such as grain yield and grain quality as well as resistance to disease and insect pests if genotypic and phenotypic data are available for a suitable training population. Multi-location data for grain yield, test weight, heading date, plant height, and powdery mildew resistance were collected for the GAWN nursery. Cross-validation results for test weight, heading date, plant height and powdery mildew resistance showed moderate to high prediction ability for these traits, which reinforces the potential of the unbalanced GAWN nursery as a training population. Furthermore, we demonstrated that for heading date, the inclusion of an additional one-year evaluation for most lines in a common environment increased the prediction ability by up to 8%. Although heritability of heading date was moderately high in this study (0.54 on a plot mean basis), it was nonetheless influenced by the year-to-year variation in winter and early spring temperatures experienced in southern USA locations. Significant genotype by environment interaction was observed for heading date (Table 2). The addition of data from a common environment did not positively affect predictive ability for plant height, as this trait was less influenced by genotype by environment interactions. Similarly, addition of data from a common environment for reaction to powdery mildew did not improve predictive ability.

Selection of training populations containing individuals more closely related with the validation set should lead to an increase in the precision of the GEBV estimates. Evaluation of population structure in our data set indicated there were two subpopulations associated with the presence or absence of the *t2BS:2GS·2GL:2BL* translocation derived from *T. timopheevii*. Thus, population differentiation was likely an artifact of the presence of a large segment of alien chromatin having many SNP in high levels of linkage disequilibrium, rather than strong population structure on the whole genome level. When the translocation was utilized as a criterion to optimize the design of the training population (Clustering method), predictive abilities were not different from the Random training population selection method. Thus, accounting for that population structure associated with the alien translocation did not improve trait prediction accuracy above the Random model. In our study, the first principal component explained only 7.6% of variation. Isidro et al. (2015) observed a slightly higher level of variation (12.7%) explained by the first principal component in a study of 1127 wheat genotypes. However, they also did not observe significant increases in model prediction ability when comparing random training population selection with clustering methods based on origin of wheat genotypes to optimize training populations. Overall, we did not find the clustering approach very useful.

In contrast, methods of training population design based on reduction of PEV mean of the validation set (PEVmean and PEVmean1) were more accurate compared with methods that selected individuals at random. This was especially true with small training population sizes, because they better accounted for the relationship between the individuals in the training population and the validation set (Habier et al. 2013). Significant increases in model predictive ability with increased population size were not observed for population size greater than 200, suggesting that not all individuals in the training population need to be utilized to get adequate levels of predictive ability in the model. Similar findings were reported with different populations of wheat (Isidro et al. 2015; Rutkoski et al. 2015), rice (Akdemir et al. 2015; Isidro et al. 2015), arabidopsis (Akdemir et al. 2015), and maize (Rincent et al. 2012; Akdemir et al. 2015). The PEVmean1 and PEVmean methods were different at training population size 100 where PEVmean had a lower model predictive ability. Akdemir et al. (2015) also found a decay or stagnation in model predictive ability for training population size of 100 individuals in a highly structured maize population when using PEVmean and Random training population selection when compared with training population of sizes ranging from 50 to 200. This result may be related with changes in the degree of genetic relationship between training populations and validation sets at smaller population sizes.

Other genomic selection studies using fixed effects associated with major QTL have been published (Arruda et al. 2016; Hoffstetter et al. 2016). Bian and Holland (2017) conclude that adding SNP associated with the trait as fixed effects in genomic predictions models yield higher predictive abilities when compared with models that only treat SNP as random effects. We used two approaches to avoid the potential bias in selecting markers for inclusion as fixed effects. For plant height and heading date, we assayed polymorphisms in major genes known to affect these traits. For powdery mildew, we identified the most significant SNP marker in each training set associated with powdery mildew resistance after masking the phenotypic data of individuals in each of the 50 different validation sets. The addition of markers as fixed effects in the genomic selection models was demonstrated to be useful for each of these traits across the complete range of training population sizes evaluated, reinforcing the utility of the addition of fixed effects in the models when available. These results agreed with Bernardo (2014) who pointed out that the predictive ability of genomic selection models can be increased by adding major genes as fixed effects when they represent a large proportion of the total variance associated with the trait under consideration (≥ 10%). However, in our study relative improvement in model predictability when adding fixed effects varied with population size. As training population size increased the influence of the fixed effects for heading date and powdery mildew in the model were reduced. The lower influence of the fixed effect in the models could be due to a greater proportion of the total variance associated with the trait explained by the random portion of the model as the number of genotypes increases.

The trend of increasing the predictive ability of the model was also observed when a combination of markers was included in the model as fixed effects. This was observed for different population sizes, and in some cases, the combination of markers outperformed the predictive ability for models with the addition of only one marker. Heading date, plant height, and powdery mildew resistance are highly heritable traits in comparison with grain yield and test weight, where the identification of a major effect QTL is complex due to the polygenic genetic architecture. The impact of adding fixed effects for these highly heritable traits was never worse than the model considering all markers as random effects. The maximum response observed was the effect of combining *Rht*-*D1* and *Rht*-*B1* for plant height with an average of 11% predictive ability increase in comparison with a model without fixed effects across all training population sizes considered.

## Conclusion

Use of historical unbalanced phenotypic data from cooperative testing among different southeastern USA breeding programs was a reliable and accurate way to incorporate genomic selection predictions into a collaborative breeding pipeline, even for a highly polygenic trait like grain yield. The training population optimization algorithm that reduced PEV increased model predictive ability for each trait analyzed, particularly for small population sizes. We demonstrated that adding markers associated with large effect genes or QTL as fixed effects in the model increased the overall model predictive ability for most training population sizes evaluated. Our results have implications for the use of training populations from 50 to 350 individuals. For all traits, minimization of PEV in the validation set and/or the addition of data for markers closely linked to or representing causal polymorphisms in genes affecting the traits had the greatest positive impact on predictive ability when training population size was between 50 and 150 individuals. In some cases, breeders may have genotyped representatives of the germplasm to be predicted and would like to target a small population of lines as a training set. In this scenario, training population optimization and targeted phenotyping of a small number of lines for expensive and/or difficult phenotyping could be done. Nonetheless, the observed predictive abilities for all training population selection methods tended to converge as training population sizes increased to 350 individuals. Even if phenotypic records for potential breeding lines are highly unbalanced across years or locations, utilizing the unbalanced data while incorporating markers associated with the trait of interest as fixed effects can lead to high prediction ability for important agronomic traits in wheat.

## Electronic supplementary material

Below is the link to the electronic supplementary material.

Supplementary material 1 (PDF 365 kb)

Supplementary material 2 (DOCX 46 kb)
