# Supplementary material for: Training population selection and use of fixed effects to optimize genomic predictions in a historical USA winter wheat panel
Source: Theor Appl Genet. 2019 Jan 24;132(4):1247–61. doi: 10.1007/s00122-019-03276-6 (PMC6449317; doi:10.1007/s00122-019-03276-6)

Supplementary Figures

S. Fig. 1 Comparison of mean predictive ability (Mean Pred. Ability) for heading date using genomic selection models with or without diagnostic markers associated with major effect QTLs as fixed effects in the model using different pre-defined training population sizes. Results are presented for training population optimization method Random (a) and PEVmean (b). Error bars represent ± one standard error of the mean

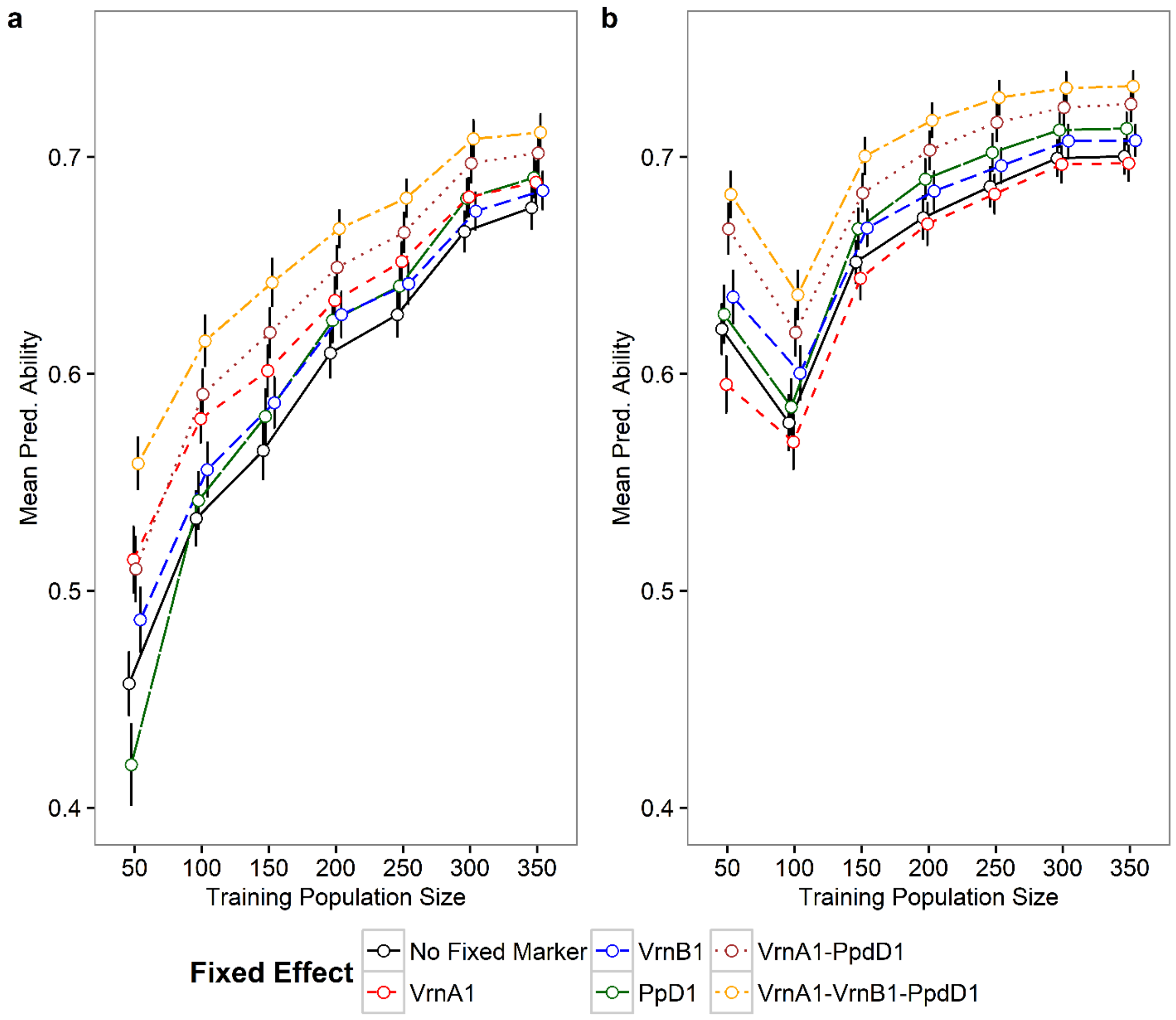

S. Fig. 2 Comparison of mean predictive ability (Mean Pred. Ability) for plant height using genomic selection models with or without diagnostic markers associated with major effect QTLs as fixed effects in the model using different pre-defined training population sizes. Results are presented for training population optimization method Random (a) and PEVmean (b). Error bars represent ± one standard error of the mean

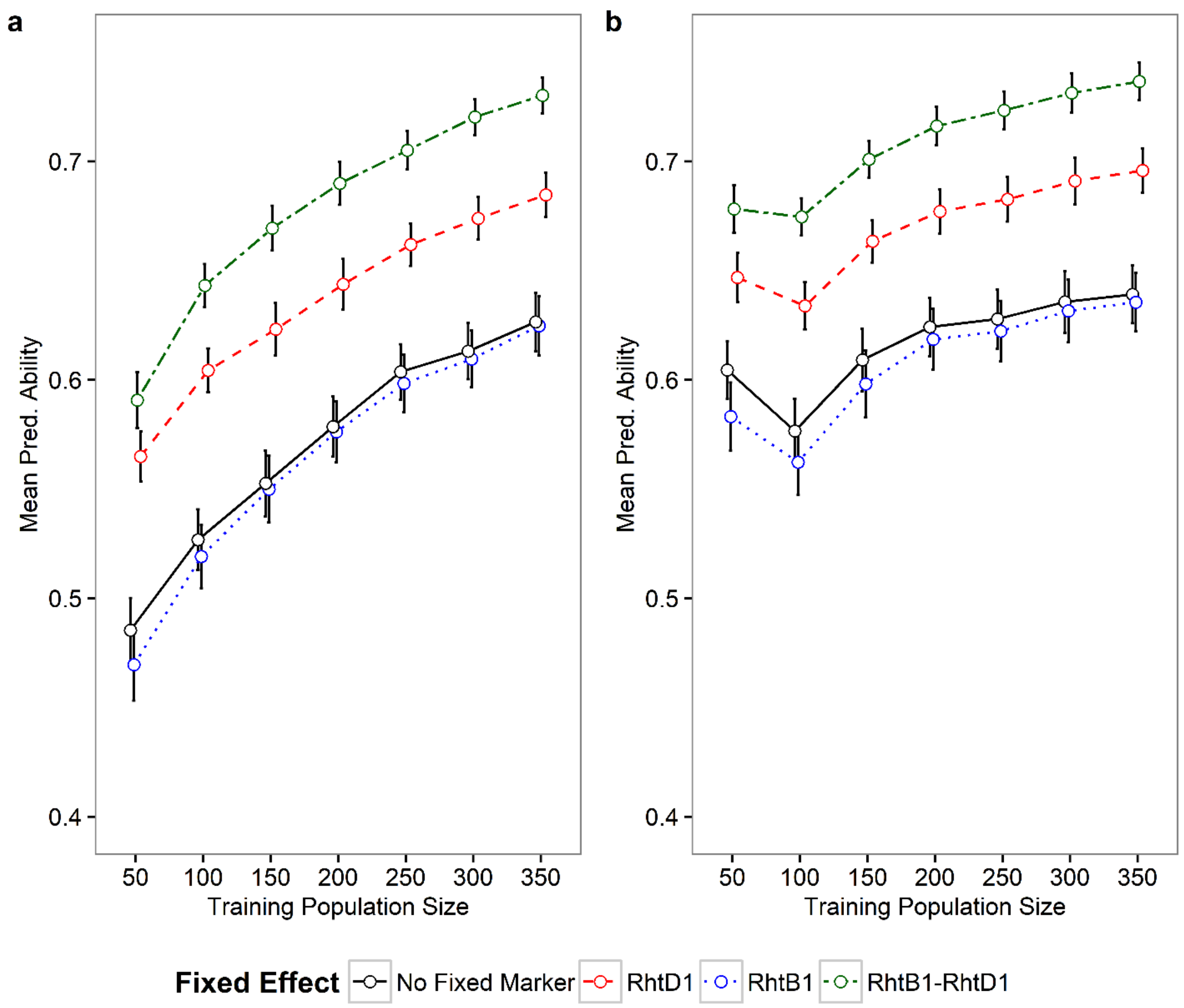

S. Fig. 3 Comparison of mean predictive ability (Mean Pred. Ability) for powdery mildew resistance using genomic selection models with or without the most significant SNP marker associated with major effect QTL as fixed effects in the model using different pre-defined training population sizes. Results are presented for training population optimization method Random (a) and PEVmean (b). Error bars represent ± one standard error of the mean

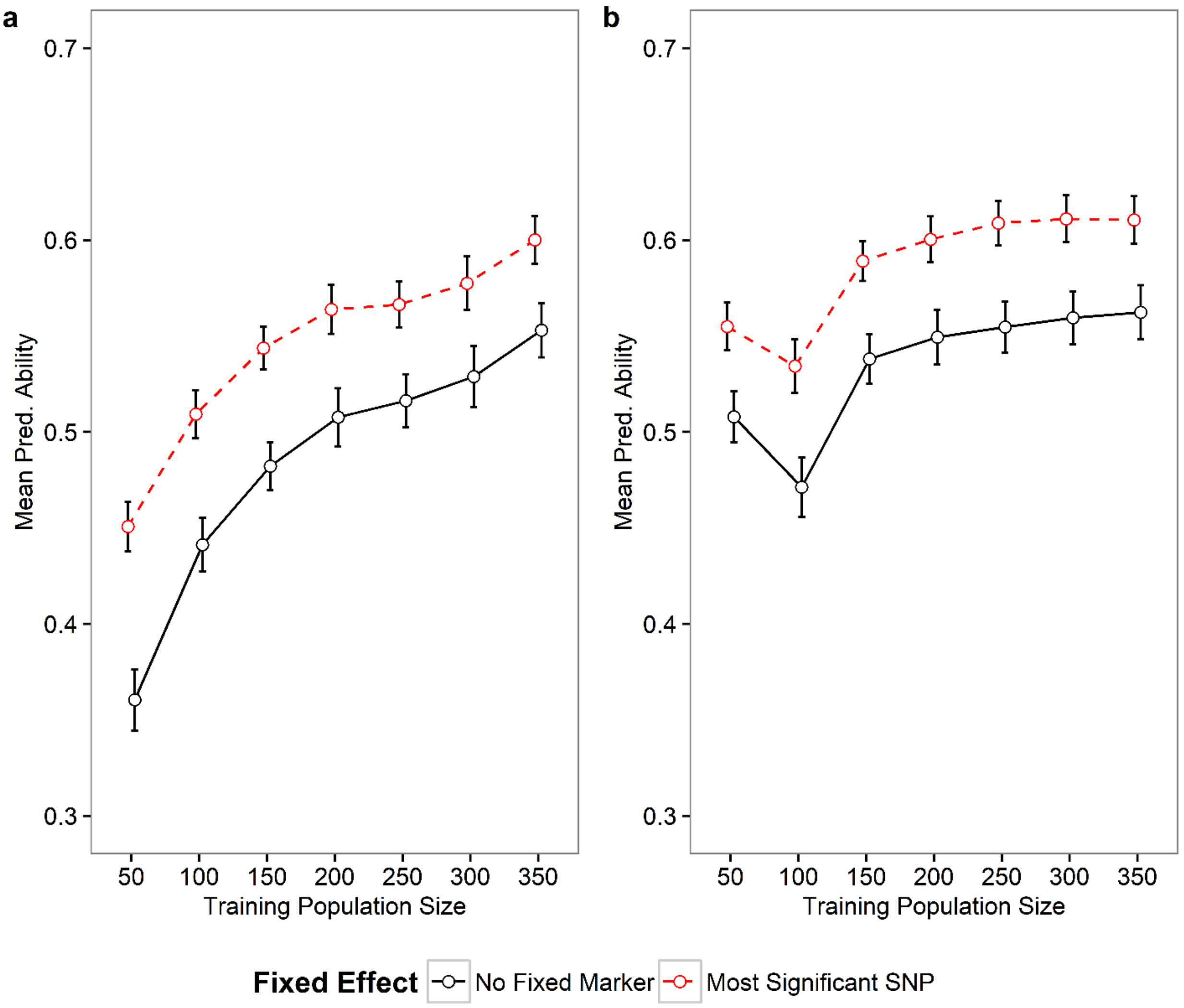

Supplement: Supplementary file 1 — Supplementary material 1 (PDF 365 kb) [file 122_2019_3276_MOESM1_ESM.pdf]
