# Supplementary material for: Training population selection and use of fixed effects to optimize genomic predictions in a historical USA winter wheat panel
Source: Theor Appl Genet. 2019 Jan 24;132(4):1247–61. doi: 10.1007/s00122-019-03276-6 (PMC6449317; doi:10.1007/s00122-019-03276-6)
Supplement: Supplementary file 2 — Supplementary material 2 (DOCX 46 kb) [file 122_2019_3276_MOESM2_ESM.docx]

**Supplementary Tables**

**S. Table 1** Number of common entries from the Gulf Atlantic Wheat Nursery evaluated for grain yield and test weight from 2008 to 2016

| YEAR | 2008 | 2009 | 2010 | 2011 | 2012 | 2013 | 2014 | 2015 | 2016 |
| --- | --- | --- | --- | --- | --- | --- | --- | --- | --- |
| 2008 | 82 | 7 | 1 | 1 | 1 | 1 | 1 | 1 | 1 |
| 2009 | 7 | 74 | 6 | 4 | 4 | 3 | 2 | 2 | 1 |
| 2010 | 1 | 6 | 75 | 10 | 4 | 3 | 2 | 2 | 1 |
| 2011 | 1 | 4 | 10 | 56 | 8 | 4 | 3 | 3 | 1 |
| 2012 | 1 | 4 | 4 | 8 | 76 | 5 | 5 | 3 | 1 |
| 2013 | 1 | 3 | 3 | 4 | 5 | 64 | 9 | 4 | 2 |
| 2014 | 1 | 2 | 2 | 3 | 5 | 9 | 38 | 8 | 2 |
| 2015 | 1 | 2 | 2 | 3 | 3 | 4 | 8 | 44 | 5 |
| 2016 | 1 | 1 | 1 | 1 | 1 | 2 | 2 | 5 | 45 |

**S. Table 2** Number of common entries from the Gulf Atlantic Wheat Nursery evaluated for plant height, heading data and reaction to powdery mildew from 2008 to 2016

| YEAR | 2008 | 2009 | 2010 | 2011 | 2012 | 2013 | 2014 | 2015 | 2016 |
| --- | --- | --- | --- | --- | --- | --- | --- | --- | --- |
| 2008 | 82 | 7 | 1 | 1 | 1 | 1 | 1 | 1 | 65 |
| 2009 | 7 | 74 | 6 | 4 | 4 | 3 | 2 | 2 | 61 |
| 2010 | 1 | 6 | 75 | 10 | 4 | 3 | 2 | 2 | 63 |
| 2011 | 1 | 4 | 10 | 56 | 8 | 4 | 3 | 3 | 52 |
| 2012 | 1 | 4 | 4 | 8 | 76 | 5 | 5 | 3 | 26 |
| 2013 | 1 | 3 | 3 | 4 | 5 | 64 | 9 | 4 | 63 |
| 2014 | 1 | 2 | 2 | 3 | 5 | 9 | 38 | 8 | 38 |
| 2015 | 1 | 2 | 2 | 3 | 3 | 4 | 8 | 44 | 35 |
| 2016 | 65 | 61 | 63 | 52 | 26 | 63 | 38 | 35 | 391 |

**S. Table 3** Complete set of location by year combination where the GAWN nursery was sown (63 total).

| Year/Location | 2008 | 2009 | 2010 | 2011 | 2012 | 2013 | 2014 | 2015 | 2016 |
| --- | --- | --- | --- | --- | --- | --- | --- | --- | --- |
| Marianna (AR) |  |  |  |  |  |  |  | X | X |
| Stuttgart (AR) | X | X | X | X | X | X | X |  |  |
| Quincy (FL) | X | X | X | X | X | X | X | X |  |
| Citra (FL) |  |  |  |  |  |  |  |  | X |
| Plains (GA) | X | X | X | X | X | X | X | X | X |
| Winnsboro (LA) | X | X | X | X | X | X | X | X | X |
| Kinston (NC) | X | X | X | X | X | X | X | X | X |
| Farmersville (TX) | X | X | X | X | X | X | X | X | X |
| Warsaw (VA) | X | X | X | X | X | X | X | X | X |

**S. Table 4** Molecular markers utilized for characterization of major genes associated with heading date, plant height and translocations

| Gene | Chromosome | Marker | Allele | Variant | Reference |
| --- | --- | --- | --- | --- | --- |
| *Ppd-A1* | 2A | *TaPpdA1_Prodel* | Deletion | Photoperiod insensitive | Nishida et al. 2013, Guedira et al. 2016 |
|  |  |  | Insertion | Photoperiod sensitive |  |
|  |  |  |  |  |  |
| *Ppd-B1* | 2B | *TaPpdBJ001* | Insertion | Photoperiod insensitive | Beales et al. 2007 |
|  |  |  | Deletion | Photoperiod sensitive |  |
|  |  |  |  |  |  |
| *Ppd-B1* | 2B | *TaPpdBJ003* | Insertion | Photoperiod insensitive | Diaz et al. 2012 |
|  |  |  | Delition | Photoperiod sensitive |  |
| *Ppd-D1a* | 2D | *TaPpdDD001* | Deletion | Photoperiod insensitive | Beales et al. 2007 |
|  |  |  | Insertion | Photoperiod sensitive |  |
|  |  |  |  |  |  |
| *Vrn-A1* | 5A | *Vrn-A1_exon4* | T/T | Long vernalization | Diaz et al. 2012 |
|  |  |  | C/C | Short vernalization |  |
|  |  |  |  |  |  |
| *Vrn-B1* | 5B | *TaVrnB1_1752* | C/C | Short vernalization | Guedira et al. 2014 |
|  |  |  | G/G | Long vernalization |  |
|  |  |  |  |  |  |
| *Rht-B1* | 4B | *Rht-B1* | T/T | *Rht-B1b* Dwarfing | Ellis et al. 2002 |
|  |  |  | C/C | Rht-B1a Wild type |  |
|  |  |  |  |  |  |
| *Rht-D1* | 4D | *Rht-D1* | T/T | *Rht-D1b* Dwarfing | Ellis et al. 2002 |
|  |  |  | G/G | Rht-D1a Wild type |  |
|  |  |  |  |  |  |
| *Translocation*  *t2AS:2NS* | 2A | *Lr37* | G/G | Lr37 Present | Helguera et al. 2003, Milus et al. 2015 |
|  |  |  | A/A | Lr37 Absent |  |
|  |  |  |  |  |  |
| *Translocation*  *t2BS:2GS·2GL:2BL* | 2B | *IWA8068* | T/T | Sr36 Present | Brown-Guedira unpublished |
|  |  |  | G/G | Sr36 Absent |  |
|  |  |  |  |  |  |
| *Translocation t1RS:1AL* | 1A | *1RS:1AL_8035* | T/T | 1RS:1AL Present | Brown-Guedira unpublished |
|  |  |  | C/C | 1RS:1AL Absent |  |
|  |  |  |  |  |  |
| *Translocation t1RS:1BL* | 1B | *1RS:1BL_6110* | A/A | 1RS:1BL Present | Brown-Guedira unpublished |
|  |  |  | G/G | 1RS:1BL Absent |  |
|  |  |  |  |  |  |

**References in table**

Beales J, Turner A, Griffiths S, Snape JW, Laurie, DA (2007) A pseudo-response regulator is misexpressed in the photoperiod insensitive *Ppd-D1a* mutant of wheat (*Triticum aestivum* L.). Theor Appl Genet, 115: 721-733.

Díaz A, Zikhali M, Turner AS, Isaac P, Laurie DA, (2012) Copy number variation affecting the Photoperiod-B1 and Vernalization-A1 genes is associated with altered flowering time in wheat (*Triticum aestivum*). PLoS One, 7: 1-11.

Ellis M, Spielmeyer W, Gale K, Rebetzke G, Richards R (2002) " Perfect" markers for the *Rht-B1b* and *Rht-D1b* dwarfing genes in wheat. Theor Appl Genet, 105: 1038-1042.

Guedira M, Maloney P, Xiong M, Petersen S, Murphy JP, Marshall D, Johnson J, Harrison S, Brown-Guedira G (2014) Vernalization duration requirement in soft winter wheat is associated with variation at the *VRN-B1* locus. Crop Sci, 54: 1960-1971.

Guedira M, Xiong M, Hao YF, Johnson J, Harrison S, Marshall D, Brown-Guedira G (2016) Heading date QTL in winter wheat (*Triticum aestivum* L.) coincide with major developmental genes VERNALIZATION1 and PHOTOPERIOD1. PLoS One, 11: 1-21.

Helguera M, Khan IA, Kolmer J, Lijavetzky D, Zhong-Qi L, Dubcovsky J (2003) PCR assays for the cluster of rust resistance genes and their use to develop isogenic hard red spring wheat lines. Crop Sci, 43: 1839-1847.

Milus EA, Lee KD, Brown-Guedira G (2015) Characterization of stripe rust resistance in wheat lines with resistance gene *Yr17* and implications for evaluating resistance and virulence. Phytopathology, 105: 1123-1130.

Nishida H, Yoshida T, Kawakami K, Fujita M, Long B, Akashi Y, Laurie DA, Kato K (2013) Structural variation in the 5′ upstream region of photoperiod-insensitive alleles *Ppd-A1a* and *Ppd-B1a* identified in hexaploid wheat (*Triticum aestivum* L.), and their effect on heading time. Mol Breed, 31: 27-37.

**S. Table 5** Single nucleotide polymorphism (SNP) distribution across the A, B, and D genomes. SNP came from the 467 genotypes genotyped using genotyping by sequencing (GBS)

|  | Genome | | |
| --- | --- | --- | --- |
| Chromosome | A | B | D |
| 1 | 1658 | 2001 | 1005 |
| 2 | 2027 | 2490 | 940 |
| 3 | 1797 | 3458 | 503 |
| 4 | 2047 | 1373 | 350 |
| 5 | 1860 | 2374 | 400 |
| 6 | 1379 | 2449 | 653 |
| 7 | 2418 | 2191 | 722 |

**S. Table 6** Mean allelic effect, allele frequency and SNP position of markers utilized as fixed effect for powdery mildew resistance

| Marker  Name | TGACv1 Scaffold | Position (bp) | N^a^ | Favorable  Allele | Unfavorable Allele | Chr | Allele  Frequency^b^ | Mean  effect ^c^ |
| --- | --- | --- | --- | --- | --- | --- | --- | --- |
| PM_SNP_1 | 560030 | 6039 | 3 | T | G | 7A | 0.11 | -0.44 |
| PM_SNP_2 | 558375 | 7138 | 1 | A | G | 7A | 0.11 | -0.37 |
| PM_SNP_3 | 556504 | 12045 | 2 | T | C | 7A | 0.11 | -0.47 |
| PM_SNP_4 | 558814 | 15364 | 11 | T | C | 7A | 0.41 | -0.45 |
| PM_SNP_5 | 556877 | 29720 | 25 | G | C | 7A | 0.12 | -0.44 |
| PM_SNP_6 | 558523 | 31200 | 8 | G | T | 7A | 0.10 | -0.46 |

^a^ Number of times the marker is the most significant in 50 different association analyses masking the phenotypes that are part of each validation set.

^b^ Frequency are indicated for the favorable/resistant allele.

^c^ The average effect of the marker estimated from 50 different training populations of size 350 selected at random.
